# Metformin Corrects Glucose Metabolism Reprogramming and NLRP3 Inflammasome-Induced Pyroptosis via Inhibiting the TLR4/NF-*κ*B/PFKFB3 Signaling in Trophoblasts: Implication for a Potential Therapy of Preeclampsia

**DOI:** 10.1155/2021/1806344

**Published:** 2021-11-11

**Authors:** Yang Zhang, Weifang Liu, Yanqi Zhong, Qi Li, Mengying Wu, Liu Yang, Xiaoxia Liu, Li Zou

**Affiliations:** Department of Obstetrics and Gynecology, Union Hospital, Tongji Medical College, Huazhong University of Science and Technology, Wuhan, China

## Abstract

NOD-like receptor family, pyrin domain-containing protein 3 (NLRP3) inflammasome-mediated pyroptosis is a crucial event in the preeclamptic pathogenesis, tightly linked with the uteroplacental TLR4/NF-*κ*B signaling. Trophoblastic glycometabolism reprogramming has now been noticed in the preeclampsia pathogenesis, plausibly modulated by the TLR4/NF-*κ*B signaling as well. Intriguingly, cellular pyroptosis and metabolic phenotypes may be inextricably linked and interacted. Metformin (MET), a widely accepted NF-*κ*B signaling inhibitor, may have therapeutic potential in preeclampsia while the underlying mechanisms remain unclear. Herein, we investigated the role of MET on trophoblastic pyroptosis and its relevant metabolism reprogramming. The safety of pharmacologic MET concentration to trophoblasts was verified at first, which had no adverse effects on trophoblastic viability. Pharmacological MET concentration suppressed NLRP3 inflammasome-induced pyroptosis partly through inhibiting the TLR4/NF-*κ*B signaling in preeclamptic trophoblast models induced via low-dose lipopolysaccharide. Besides, MET corrected the glycometabolic reprogramming and oxidative stress partly via suppressing the TLR4/NF-*κ*B signaling and blocking transcription factor NF-*κ*B1 binding on the promoter PFKFB3, a potent glycolytic accelerator. Furthermore, PFKFB3 can also enhance the NF-*κ*B signaling, reduce NLRP3 ubiquitination, and aggravate pyroptosis. However, MET suppressed pyroptosis partly via inhibiting PFKFB3 as well. These results provided that the TLR4/NF-*κ*B/PFKFB3 pathway may be a novel link between metabolism reprogramming and NLRP3 inflammasome-induced pyroptosis in trophoblasts. Further, MET alleviates the NLRP3 inflammasome-induced pyroptosis, which partly relies on the regulation of TLR4/NF-*κ*B/PFKFB3-dependent glycometabolism reprogramming and redox disorders. Hence, our results provide novel insights into the pathogenesis of preeclampsia and propose MET as a potential therapy.

## 1. Introduction

Preeclampsia (PE), characterized by new-onset hypertension typically after 20 weeks of gestation together with multisystem involvement, complicates 2%–8% of pregnancies globally with huge associated healthcare burdens to the whole of society [1]. Although the precise pathogenesis of PE remains elusive, pyroptosis, a newly discovered programmed cell death process accompanied by sterile inflammatory cascades, has now been proved existent in the preeclamptic placentas while its regulatory mechanism remains vague [2, 3].

Pyroptosis is efficiently controlled by the NOD-like receptor family, pyrin domain-containing protein 3 (NLRP3) inflammasome [4] composed of NLRP3, caspase 1, and apoptosis-associated speck-like protein containing a caspase recruitment domain (ASC). NLRP3 inflammasome generates interleukin- (IL-) 1*β* and IL-18 and maturates gasdermin D (GSDMD) to induce a lytic form of cell death, pyroptosis [5]. Toll-like receptor 4 (TLR4), a crucial receptor of exogenous pathogen-associated molecular patterns (PAMPs) and endogenous damage-associated molecular patterns (DAMPs) [6], has been widely known to participate in cellular pyroptosis through activating its canonical downstream nuclear factor-*κ*B (NF-*κ*B) signaling [7, 8]. The NF-*κ*B family of transcription factors, consisting of five Rel-homology-containing proteins (cRel, RelA, RelB, NF-*κ*B1, and NF-*κ*B2), forms numerous NF-*κ*B dimers normally binding to inhibitors of NF-*κ*B (I*κ*B) in the cytoplasm. The phosphorylation of I*κ*B kinase (IKK) contributes to the phosphorylation and degradation of I*κ*B, resulting in the release and nuclear translocation of the NF-*κ*B family of transcription factors, finally modulating the transcription of downstream genes, such as NLRP3 [9, 10]. Extensive research has reported the vital role of highly expressed and overactivated TLR4/NF-*κ*B signaling in the preeclamptic placentas via causing trophoblastic inflammation, oxidative stress, and dysfunction [11, 12]. With the increased expression of NLRP3 and related mediators (caspase 1 and IL-1*β*) found in the placentas [13, 14], trophoblastic pyroptosis has now been suggested to be one of the PE pathogenesis [2, 3]. However, it remains to be verified whether the overactive TLR4/NF-*κ*B signaling in preeclamptic trophoblasts triggers the NLRP3 inflammasome-induced pyroptosis.

Intriguingly, the TLR4/NF-*κ*B signaling is closely related to cellular metabolic reprogramming. The activation of the TLR4/NF-*κ*B signaling leads to the preferential use of glycolysis, instead of mitochondrial oxidative phosphorylation (OXPHOS) in macrophages and dendritic cells [15–17]. Regrettably, the metabolic characters of the preeclamptic placentas remain indefinite. Reportedly, the energy required to support the physiological activities of trophoblasts relies principally on glycometabolism [18]. There have been plenty of reports about mitochondrial destruction and dysfunction in the preeclamptic placentas [19]. The latest research has further claimed that preeclamptic trophoblasts are very likely to undergo the glycometabolic reprogramming to glycolysis [20]. Nonetheless, whether the overactive uteroplacental TLR4/NF-*κ*B signaling reprograms glycometabolism in the preeclamptic placentas remains to be settled.

Recently published studies have suggested that NLRP3 inflammasome-induced pyroptosis can be modulated by some key glycolytic enzymes [21], such as hexokinase 1 (HK1) and M2 isoform of pyruvate kinase (PKM2). The inhibition of HK1 and PKM2 not only suppressed glycolysis flux but also mitigated NLRP3 inflammasome-activated pyroptosis [22, 23]. 6-Phosphofructo-2-kinase/fructose-2,6-bisphosphatase 3 (PFKFB3), a potent glycolytic regulator, accelerates glycolytic flux by promoting fructose-2,6-bisphosphate (F2,6P2) synthesis and facilitating the allosteric activation of 6-phosphofructo-1-kinase 1 (PFK1) [24]. PFKFB3 is expressed at a certain level in the normal placentas. However, we have previously reported that the PFKFB3 expression in the preeclamptic placentas was significantly increased, modulating the trophoblastic inflammation and oxidative stress via the NF-*κ*B signaling [25]. Thus, it needs to be further clarified whether the trophoblastic glycolysis represented by PFKFB3 regulated the NLRP3 inflammasome-induced pyroptosis at the uteroplacental interface.

Up to now, there has been a clinical dilemma that current clinical therapies for PE are mainly symptomatic treatments, while, thus far, the only curative intervention for PE is the delivery of the fetus and placenta, albeit with raised consequent risks [26]. Hence, more possible therapeutic strategies remain to be further explored. Metformin (MET), the first-line therapy for type II diabetes, has long been clinically used for adjusting glycometabolism [27]. Surprisingly, randomized clinical trials, as well as meta-analyses, have reported the preventive and therapeutic effects of MET on PE while the underlying mechanism remains unclear [28, 29]. Previous studies have pointed out that MET effectively inhibits the TLR4/NF-*κ*B signaling [30–32]. Recent studies have reported that MET prevents trophoblastic apoptosis caused by insulin toxicity [33] as well as alleviates pyroptosis in ischemia-reperfusion injuries [34, 35]. Moreover, the safety of taking MET during human pregnancies has been preliminarily ensured [36]. Thus, the therapeutic potential of MET in PE is worth in-depth study [26].

In the present study, we hypothesized that the TLR4/NF-*κ*B/PFKFB3 pathway may be a novel link between metabolism reprogramming and NLRP3 inflammasome-induced pyroptosis in trophoblasts. Further, MET alleviates the NLRP3 inflammasome-induced pyroptosis, which partly relies on the regulation of TLR4/NF-*κ*B/PFKFB3-dependent glycometabolism reprogramming and redox disorders. This research may provide novel insights into the pathogenesis of preeclampsia and propose MET as a potential therapy.

## 2. Materials and Methods

### 2.1. Cell Culture

The immortalized human extravillous trophoblast cell line HTR-8/SVneo was a kind gift from Dr. Charles Graham (Queen's University, Canada) [25]. Cells were cultured in RPMI 1640 medium (Hyclone) containing 10% fetal bovine serum (Gibco) and 100 U/ml penicillin-streptomycin solutions (PYG0016, Boster), under a 5% CO_2_ atmosphere at 37°C.

### 2.2. Reagents and Treatment

HTR-8/SVneo cells were cultured with lipopolysaccharide (LPS, 200 ng/ml, Sigma) for 24 h to construct the preeclamptic trophoblast model as previously reported [10, 37–40]. 10~40 *μ*M MET (S1950, Selleck Chemicals) were used for intervention. HTR-8/SVneo cells were pretreated with 3-(3-pyridinyl)-1-(4-pyridinyl)-2-propen-1-one (3PO, 10 *μ*M, Merck Millipore) for 6 h to inhibit PFKFB3.

### 2.3. Cell Viability Assay

The trophoblastic viability was detected via the CCK8 assay (C0038, Beyotime). HTR8/SVneo cells were seeded at a density of 5 × 10^3^ cells/well on 96-well plates. The cellular viability was determined at 24 h after being induced by MET. The CCK8 solution (10 *μ*l) was added to each well, followed by 1 h of incubation at 37°C in the dark. The absorbance at 450 nm was measured using a Multimode Reader (Infinite F50, Tecan).

### 2.4. Apoptosis Analysis

The trophoblastic apoptosis was assessed using an Annexin V-FITC Apoptosis Detection Kit (BD Biosciences Pharmingen). Briefly, after the indicated treatments, the cells were resuspended in binding buffer and stained with 5 *μ*l of propidium iodide (PI) and 5 *μ*l of Annexin V-FITC. Subsequently, the stained cells were evaluated using a flow cytometer (BD Biosciences Pharmingen), and the percentage of apoptotic HTR-8/SVneo cells was Q2 plus Q3.

### 2.5. Cell Transfection

The pEX-1 vector (GenePharma, Shanghai, China) was used for PFKFB3 overexpression. The pEX-1 vector (Genechem, Shanghai, China) was used for TLR4 and NF-*κ*B1 overexpression. The empty plasmid vector was used as the control. These plasmids were transfected into cells using Lipofectamine 3000 Reagent (Thermo Fisher Scientific) as required. The cells were transfected for 48 h and then collected for subsequent analyses. The transfection efficiency was evaluated using western blotting.

### 2.6. Western Blot Analysis

Proteins were extracted using radioimmunoprecipitation buffer and 100× phenylmethylsulfonyl. The protein concentration was measured by a BCA protein assay kit (P0010S, Beyotime). Sixty micrograms of protein per sample were subjected to 10% sodium dodecyl sulfate-polyacrylamide gel electrophoresis and transferred onto polyvinylidene fluoride membranes (0.45 *μ*m pore size, Millipore). Then, the membranes were blocked with 5% nonfat milk in Tris-buffered saline containing 0.05% Tween 20 (TBST) for 1 h at room temperature. Next, the membranes were incubated, respectively, with TLR4 antibody (sc-293072, Santa Cruz), PFKFB3 antibody (13763-1-AP, Proteintech), NF-*κ*B1 antibody (14220-1-AP, Proteintech), NF-*κ*B p65 antibody (#8242, Cell Signaling Technology), phosphoNF-*κ*B p65 antibody (#3033, Cell Signaling Technology), I*κ*B antibody (A11397, ABclonal), phosphoI*κ*B antibody (AP0614, ABclonal), phosphoIKK antibody (AP0891, ABclonal), NLRP3 antibody (19771-1-AP, Proteintech), caspase-1 antibody (22915-1-AP, Proteintech), ASC antibody (10500-1-AP, Proteintech), GSDMD antibody (20770-1-AP, Proteintech), and superoxide dismutase 2 (SOD2) antibody (A1340, ABclonal) overnight at 4°C. *β*-Actin antibody (20536-1-AP, Proteintech) was used as an internal control. The next day, they were brought to room temperature and washed with TBST three times. Then, the bands were incubated with appropriate secondary antibodies (1: 4,000, Affinity Biosciences) at room temperature for 1 h and visualized using an ECL kit (Millipore) according to the manufacturer's recommendations.

### 2.7. Enzyme-Linked Immunosorbent Assay (ELISA)

Culture supernatant contents of IL-1*β* (HM10206, Bioswamp) and IL-18 (HM10337, Bioswamp) were assessed via the ELISA kits following the manufacturer's instructions.

### 2.8. Hoechst 33342/PI Double-Fluorescent Staining

Cellular pyroptosis was assessed using Hoechst 33342/PI double-fluorescent staining [41]. HTR-8/SVneo cells were cultured in six-well plates at a density of 5 × 10^5^ cells per well, and the cells were transfected with varying constructs or treated with drugs. Next, the cells were stained with 10 *μ*l Hoechst 33342 (C1027, Beyotime) solution at 37°C in the dark for 10 minutes, followed by staining with 5 *μ*l PI at 25°C in the dark for 15 minutes. The stained cells were observed under a confocal microscope.

### 2.9. Electron Microscopy

For transmission electron microscope (TEM) observations, the samples were fixed in 4% paraformaldehyde (Sigma) and 2.5% glutaraldehyde (Sigma) in 0.1 M phosphate (Sigma) buffer overnight. After washing in 0.1 M phosphate buffer, the samples were postfixed for 1 h in 1% osmium tetroxide (Sigma) prepared in the same buffer. The samples were dehydrated with a graded series of ethyl alcohol concentrations, embedded in Epon 812, and polymerized at 60°C for 3 days. Ultrathin sections (60–70 nm) were obtained using an ultramicrotome (Leica Ultracut UCT, Germany). Ultrathin sections collected on grids (200 mesh) were examined in TEM (JEM 1010) operating at 60 kV, and images were recorded by a charge-coupled device camera (SC1000, Gatan). The images from electron microscopy were analyzed and measured by the ImageJ software for calculating the longest axis of mitochondrial length. At least over fifty mitochondria were measured and analyzed per sample to obtain data [42].

### 2.10. Mitochondrial Membrane Potential (MMP)

MMP was evaluated via fluorescent probe JC-1 (C2006, Beyotime) using a confocal microscope. Normal mitochondria were presented as red while mitochondria with decreased MMP were presented as green. The ratio of green/red fluorescence intensity was calculated by ImageJ software to assess the MMP changes. At least 6 images per condition were analyzed. MMP was also evaluated by a flow cytometer. HTR-8/SVneo cells with healthy mitochondria were distributed in Q2, while cells with MMP decline were distributed in Q3.

### 2.11. Extracellular Acidification Rate (ECAR) and Oxygen Consumption Rate (OCR)

For OCR and ECAR measurement, HTR-8/SVneo cells were seeded into Seahorse 24-well plates and were then treated according to the manufacturer's protocol after the indicated treatment. Seahorse Xfe24 analyzer (Seahorse Bioscience, Boston, MA, United States) was used for evaluation. All results were normalized to the cell number. For OCR measurement, oligomycin, carbonyl cyanide 4-(trifluoromethoxy) phenylhydrazone (FCCP), and rotenone were added. Basal respiration = (last rate measurement before oligomycin injection) − (minimum rate measurement after rotenone injection). Maximal respiration = (maximum rate measurement after FCCP injection) − (minimum rate measurement after rotenone injection). For ECAR evaluation, glucose, oligomycin, and 2-deoxyglucose (2-DG) were added according to the manufacturer's instructions. Glycolysis = (last rate measurement before oligomycin injection) − (minimum rate measurement before glucose injection). Glycolytic capacity = (maximum rate measurement after oligomycin injection) − (minimum rate measurement after 2 − DG injection).

### 2.12. Immunofluorescent Staining

Immunofluorescence in regards to the frozen placental tissues of the third trimester, detection of PFKFB3 activity in the normotensive and preeclamptic placenta was performed as described previously [25]. Briefly, the primary antibodies were diluted in the appropriate blocking solution at the following concentrations: PFKFB3 antibody (1: 100; 13763-1-AP, Proteintech) and mouse anti-CK7 (1: 100; ab68459, Abcam). The secondary antibody was a FITC-labeled goat anti-rabbit IgG (Beyotime) and a Cy3-labeled goat anti-mouse IgG (Beyotime). The nuclei were stained with DAPI (Beyotime) for 3 min. HTR-8/SVneo cells in different groups cultured on coverslips were fixed by 4% paraformaldehyde for 20 min and then extracted with 0.5% Triton X-100 solution for 5 minutes. After blocking with TBST containing 1% bovine serum albumin, cells were incubated with indicated primary antibody NF-*κ*B1 (1: 100; 14220-1-AP, Proteintech) for 1 h. After that, cells were washed and incubated with FITC-labeled goat anti-rabbit IgG (Beyotime) for 1 h, following with DAPI for 3 min. Images were captured with an inverted microscope and were analyzed by ImageJ.

### 2.13. Measurements of the Lactate Concentration, ATP Concentration, and NADPH/NADP+ Ratio

Extracellular lactate, intracellular ATP, and intracellular NADPH levels were measured using the following assay kits, according to the manufacturers' instructions: lactate assay kit (A019-2-1, Nanjing Jiancheng), ATP assay kit (S0027, Beyotime), and NADPH/NADP+ ratio (ab65349, Abcam).

### 2.14. Intracellular ROS Detection

Intracellular ROS generation was determined by fluorescent probe DCFH-DA (S0033, Beyotime). After washing twice with cold PBS, the HTR-8/SVneo cells were incubated with 10 *μ*M DCFH-DA in the dark for 20 min. Green fluorescent images were captured using a confocal microscope. The level of intracellular ROS was also detected by flow cytometry. ImageJ and FlowJo software were used to analyze the results.

### 2.15. Chromatin Immunoprecipitation (ChIP) Assay

ChIP assays were conducted using the EZ-ChIP™ kit (Millipore). Briefly, protein-DNA complexes were cross-linked by 1% formaldehyde then quenched using 125 mM glycine. Cells were collected in shearing buffer (Diagenode, Denville, NJ, USA), and chromatin was sheared to an average DNA fragment size of 0.5-1 kb using a Bioruptor sonicator (Diagenode). After centrifugation, the supernatant was incubated with IgG or specific antibodies for NF-*κ*B1, and chromatin DNA was purified and subjected to PCR detection. Primers for the PFKFB3 promoter are sense primer 5′-ATTGGCTGCTTTCATAGACCC-3′ and anti-sense primer 5′-CCAGGCTCAACCCATACTCC-3′.

### 2.16. Luciferase Reporter Gene Assays

Briefly, NF-*κ*B1 response peak (p65 RE) and either wildtype or mutated PFKFB3 luciferase reporter vectors (containing a mutation in any of the predicted p65 binding sites) were transfected into the HTR-8/SVneo cells. After a 48 h incubation, the relative luciferase activity was measured with a dual-luciferase reporter system (RG027, Beyotime) using a Multimode Reader (Infinite M1000, Tecan).

### 2.17. RNA Extraction and qRT-PCR

Total RNA was isolated from the cells using RNAiso Plus (Takara, Japan) and then reversely transcribed using PrimeScript 1st RT Master Mix (Takara) according to the instructions of the manufacturer. The primer sequences were as follows: *β*-actin (internal group) forward primer: 5′-CACGATGGAGGGGCCGGACTCATC-3′; reverse primer: 5′-TAAAGACCTCTATGCCAACACAGT-3′ and PFKFB3 forward primer: 5′-ATTGCGGTTTTCGATGCCAC-3′; reverse primer: 5′-GCCACAACTGTAGGGTCGT-3′. Quantitative real-time polymerase chain reaction (qRT-PCR) was performed on StepOne Real-Time PCR System with SYBR Premix Ex Taq (Takara) as described by the manufacturer's instructions. The qRT-PCR reaction consisted of a 95°C denaturation step for 30 s, 40 cycles (95°C for 5 s, 65°C for 30 s, and 60°C for 45 s) and an extension at 72°C for 60 s. The expression of target genes was established using the comparative cycle threshold (Ct; 2^−ΔΔCT^) method.

### 2.18. Ubiquitination Assays

Ubiquitination assays were performed as described previously [43]. To assess the ubiquitination status of NLRP3, cells were collected in NETN buffer [20 mM Tris-HCl (pH 8), 100 mM NaCl, 1 mM EDTA, 0.5% Nonidet P-40, protease inhibitor mixture, and 1 mM phenylmethylsulfonyl fluoride], treated with 1% SDS, heated to 95°C for 15 min, and then diluted 10-fold in lysis buffer. The precleared lysates were incubated with the NLRP3 antibody (2 *μ*g) in the presence of 40 *μ*l of protein A/G agarose overnight at 4°C. Immunoprecipitates were collected by centrifugation for 1 min at 1000 × *g* at 4°C. The beads were then washed four times with lysis buffer. An aliquot (40 *μ*l) of SDS-PAGE sample buffer (62.5 mM Tris-HCl, pH 6.8, 10% glycerol, 2% SDS, 0.001% bromophenol blue, and 10% *β*-mercaptoethanol) was added to the beads. Samples were resolved by SDS-PAGE, transferred to nitrocellulose membranes, and analyzed by immunoblotting with anti-ubiquitin antibody (1: 100, #3936, CST).

### 2.19. Statistical Analyses

For all analyses, data are shown as the mean ± standard deviation. The Student *t*-test was used for comparing two samples, and multigroup comparison was carried out using one-way ANOVA followed by Tukey's post hoc test. Statistical analysis was performed using GraphPad Prism 8.0 (USA). The *P* value of less than 0.05 was considered statistically significant. All data were obtained from ≥3 independent experiments.

## 3. Results

### 3.1. Pharmacological MET Concentrations Alleviated LPS-Induced NLRP3 Inflammasome Formation and Trophoblastic Pyroptosis

**Figure 1:**
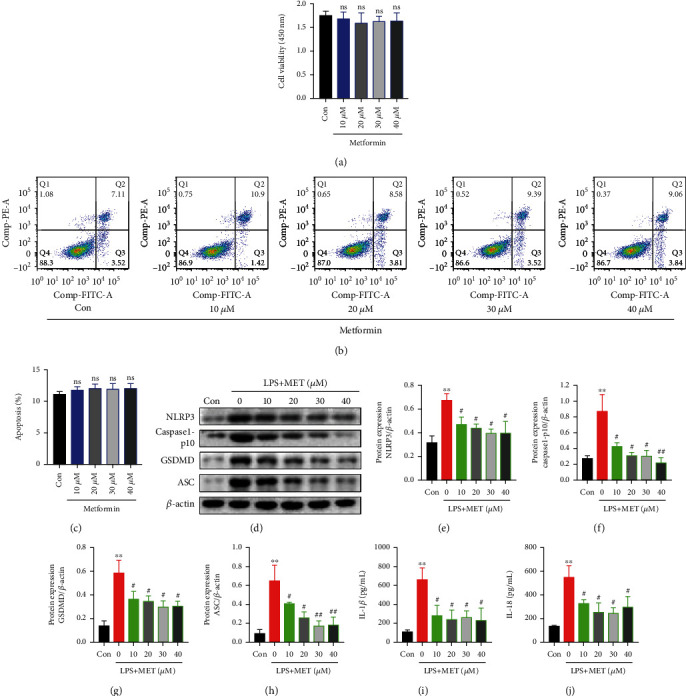
Pharmacological MET concentrations alleviated LPS-induced NLRP3 inflammasome formation and trophoblastic pyroptosis. (a) CCK-8 assays of HTR-8/SVneo cells treated with the indicated concentration of MET for 24 h. (b, c) Flow cytometry assays and quantitative analysis were performed to determine the apoptotic rates in HTR-8/SVneo cells treated with the indicated MET concentration for 24 h. (d–h) Western blot analysis of NLRP3, caspase1-p10, GSDMD, and ASC protein expression and densitometry quantification of NLRP3 (e), caspase1-p10 (f), GSDMD (g), and ASC (h) levels in HTR-8/SVneo cells were cultured with 200 ng/ml LPS and MET at different concentrations, respectively (10, 20, 30, and 40 *μ*M). (i, j) ELISA analysis of IL-1*β* (i) and IL-18 (j) concentrations in cell culture medium. Data are shown as the mean ± SD from three independent experiments. ns: not significant compared with the Con group. ^∗∗^*P* < 0.01 compared with the Con group. ^#^*P* < 0.05 compared with the LPS group. ^##^*P* < 0.01 compared with the LPS group by Student's *t*-test. SD: standard deviation.

According to the MET pharmacokinetics in pregnancy [26, 44], we carefully designed a pharmacological MET concentration gradient (10~40 *μ*M) in our study, which had no effects on trophoblastic viability or apoptosis through CCK-8 assays (Figure 1(a)) and flow cytometry analysis (Figures 1(b) and 1(c)). Low-dose LPS has long been utilized to induce preeclamptic trophoblast models [39, 40]. In line with the previous report [37], LPS (200 ng/ml, 24 h) in our study significantly upregulated the expressions of NLRP3, caspase1-p10, ASC, and GSDMD proteins (Figures 1(d)–1(h)), as well as the secretion of IL-1*β* and IL-18 (Figures 1(i) and 1(j)) compared with the normal control. However, in our study, pharmacological MET concentrations downregulated NLRP3 expression and those downstream molecules in a dose-dependent manner (Figures 1(d)–1(j)). Given the previous studies and our data [33], 10 *μ*M MET was chosen for subsequent experiments.

### 3.2. Pharmacological MET Concentration Suppressed NLRP3 Inflammasome-Induced Pyroptosis Partly through Inhibiting the TLR4/NF-*κ*B Signaling

**Figure 2:**
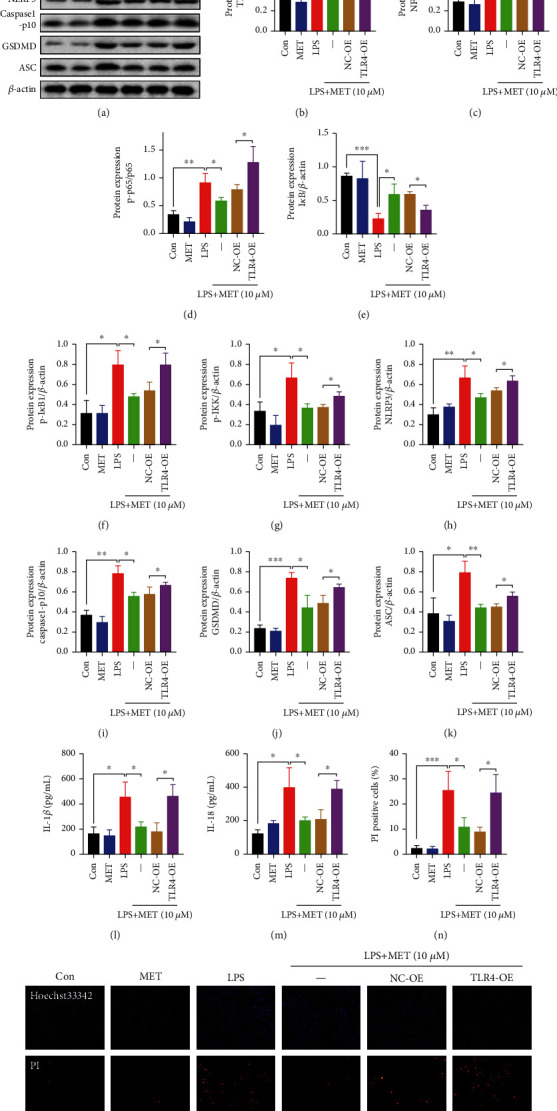
Pharmacological MET concentration suppressed NLRP3 inflammasome-induced pyroptosis partly through inhibiting the TLR4/NF-*κ*B signaling. HTR-8/SVneo cells were transfected with TLR4 plasmid for overexpression or negative vector; 200 ng/ml LPS and 10 *μ*M MET were subsequently incubated. (a–k) Western blot analysis of TLR4, NF-*κ*B1, p65, p-p65, I*κ*B, p-I*κ*B, p-IKK, NLRP3, caspase1-p10, GSDMD, and ASC protein expression and densitometry quantification of TLR4 (b), NF-*κ*B1 (c), p-p65/p65 (d), I*κ*B (e), p-I*κ*B (f), p-IKK (g), NLRP3 (h), caspase1-p10 (i), GSDMD (j), and ASC (k) levels. (l, m) ELISA analysis of IL-1*β* (l) and IL-18 (m) concentrations in cell culture medium from different groups. (n, o) Representative immunofluorescence images and quantification of double-fluorescent staining with PI (red) and Hoechst33342 (blue). Scale bar: 100 *μ*m. Data are shown as the mean ± SD from three independent experiments. ^∗^*P* < 0.05, ^∗∗^*P* < 0.01, and ^∗∗∗^*P* < 0.001 by Student's *t*-test. SD: standard deviation; NC-OE: negative vector; TLR4-OE: TLR4 overexpression plasmid.

Firstly, we validated the efficiency of TLR4 overexpression plasmid after 48 h transfection by western blot analysis (Figure S1A). Compared with the normal control, LPS markedly exacerbated its recognized downstream NF-*κ*B signaling, characterized by the increased protein expression of TLR4 and NF-*κ*B1, phosphorylation states of NF-*κ*B and p65 (p-p65), phosphorylation states of I*κ*B (p-I*κ*B), phosphorylation states of IKK (p-IKK), and the reduced expression of I*κ*B (Figures 2(a)–2(g)). Nevertheless, MET treatment partly diminished all those effects, indicating that MET is an effective inhibitor of the TLR4/NF-*κ*B signaling [45] (Figures 2(a)–2(g)). We further induced TLR4 overexpression to reactivate its canonical downstream NF-*κ*B signaling (Figures 2(a)–2(g)), which upregulated the protein levels of NLRP3, caspase1-p10, ASC, and GSDMD (Figures 2(h)–2(k)) and enhanced IL-1*β* and IL-18 release (Figures 2(l) and 2(m)) again. Hoechst33342/PI staining experiment showed increased PI uptake in LPS-stimulated trophoblasts (Figures 2(n) and 2(o)). The amounts of the PI-stained cells were markedly reduced after MET treatment, which was partly reversed by TLR4 overexpression (Figures 2(n) and 2(o)). Hence, we verified that the TLR4/NF-*κ*B signaling contributed to NLRP3 inflammasome-induced pyroptosis in trophoblasts while MET suppressed trophoblastic pyroptosis partly through inhibiting the TLR4/NF-*κ*B signaling.

### 3.3. Pharmacological Concentration of MET Corrected Metabolic Reprogramming in Trophoblasts Partly via the Suppression of the TLR4/NF-*κ*B Signaling

**Figure 3:**
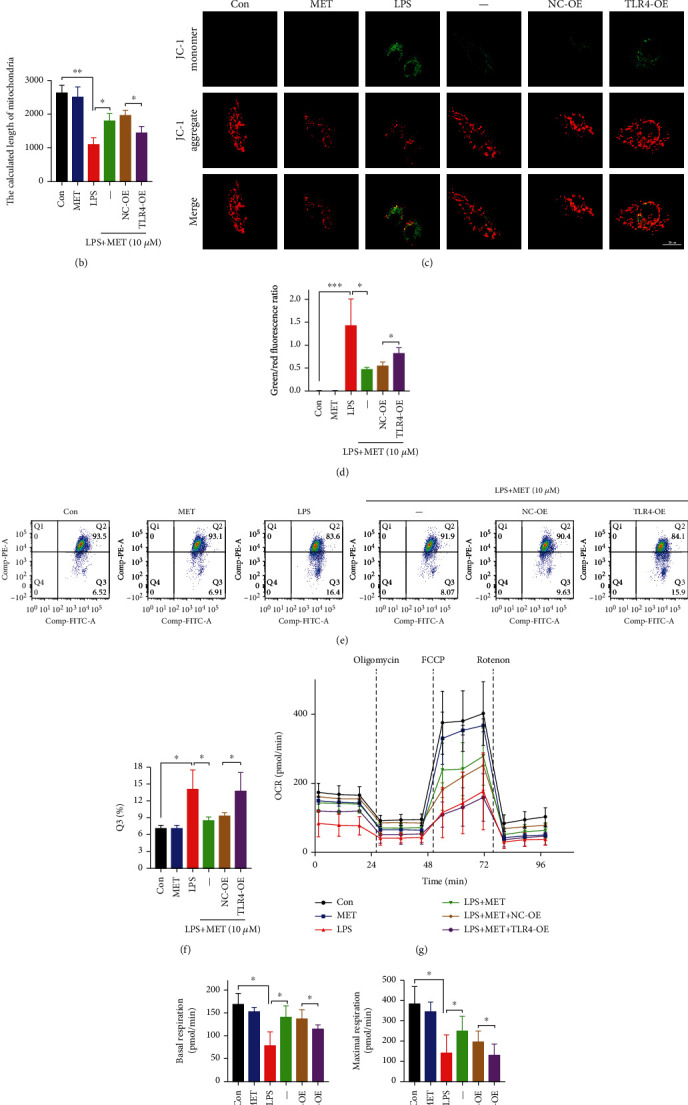
Pharmacological concentration of MET preserved mitochondrial homeostasis in trophoblasts partly via the suppression of the TLR4/NF-*κ*B signaling. HTR-8/SVneo cells were transfected with TLR4 plasmid for overexpression or negative vector; 200 ng/ml LPS and 10 *μ*M MET were subsequently incubated. (a, b) HTR-8/SVneo cells in different groups were observed by a transmission electron microscope to evaluate the mitochondrial structure, and the mitochondrial length was analyzed quantitatively. Scale bar: 500 nm. (c, d) MMP was evaluated by a confocal microscope. The ratio of green/red puncta was calculated to assess the MMP changes. At least 6 images per condition were analyzed. Scale bar: 20 *μ*m. (e, f) Flow cytometry assays and quantitative analysis were also performed to determine the MMP of HTR-8/SVneo cells in different groups. (g–i) The OCR assay was used to observe the mitochondrial respiratory function. Data are shown as the mean ± SD from three independent experiments. ^∗^*P* < 0.05, ^∗∗^*P* < 0.01, and ^∗∗∗^*P* < 0.001 by Student's *t*-test. SD: standard deviation; NC-OE: negative vector; TLR4-OE: TLR4 overexpression plasmid.

The mitochondrial was long cable-like, thin strips in normal trophoblasts. On TLR4/NF-*κ*B activation, the trophoblastic mitochondria were divided into several fragments with mitochondrial swell and cristae damage, suggesting the mitochondrial structure disruption. Despite under LPS stimulation, mitochondria in the MET-treated cells showed a predominantly elongated form, which was partially destroyed by TLR4 overexpression, together with the swollen and broken cristae (Figures 3(a) and 3(b)). Mitochondrial membrane potential (MMP) assays via JC-1 fluorescence staining showed that LPS decreased the trophoblastic MMP while MET partly recovered it, although this effect was blocked by TLR4 overexpression (Figures 3(c) and 3(d)). Consistent results were also obtained from the JC-1 flow cytometry analysis (Figures 3(e) and 3(f)). OCR assays of OXPHOS were then conducted, further confirming that the OXPHOS was damaged on TLR4/NF-*κ*B activation. The basal and maximum respiration rates were both improved after MET exposure, and this effect was blocked by TLR4-overexpression again (Figures 3(g)–3(i)).

**Figure 4:**
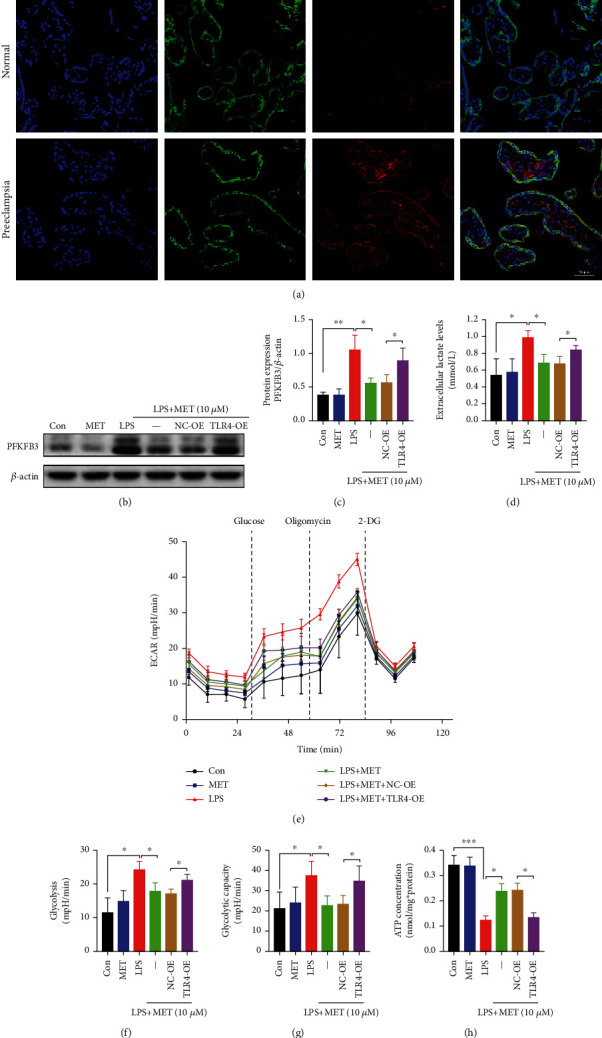
Pharmacological concentration of MET suppressed overactive glycolysis in trophoblasts partly via inhibiting the TLR4/NF-*κ*B signaling. (a) Double labeling the immunofluorescence analysis of PFKFB3 (red) protein expression and localization in the placentae from full-term normal pregnancies and preeclampsia patients. DAPI (blue) and CK7 (green) for trophoblast localization. Scale bar: 50 *μ*m. (b, c) Western blot analysis of PFKFB3 protein expression and the densitometry quantification. (d) Extracellular lactate release determination. (e–g) Glycolysis and glycolysis capacity were both detected by ECAR assay. (h) Cellular ATP concentration determination. Data are shown as the mean ± SD from three independent experiments. ^∗^*P* < 0.05, ^∗∗^*P* < 0.01, and ^∗∗∗^*P* < 0.001 by Student's *t*-test. SD: standard deviation; NC-OE: negative vector; TLR4-OE: TLR4 overexpression plasmid.

Previously, we have reported that PFKFB3 is highly expressed in the preeclamptic placentas [25]. In this study, double labeling of immunofluorescence further demonstrated that PFKFB3 protein expressed in trophoblast cells was significantly increased in the preeclamptic trophoblasts (Figure 4(a)). In line with the latest report, LPS elevated PFKFB3 protein levels in this study, which was alleviated by pharmacological MET concentration [46]; however, TLR4 overexpression increased PFKFB3 expression again (Figures 4(b) and 4(c)). In lactate assays of culture medium, LPS significantly enhanced lactate release while we observed weaker lactate secretion in the LPS+MET group; however, TLR4 overexpression increased lactate secretion again (Figure 4(d)). Furthermore, measurement of ECAR revealed an abnormal increase in glycolysis and glycolysis capacity in the LPS group compared with the normal control group. This effect was partially abolished by MET treatment, though the TLR4 overexpression upregulated the glycolytic levels again (Figures 4(e)–4(g)). Eventually, trophoblast cells cultured with LPS exhibited ATP shortages despite the increased capacity for glycolysis, which was partially restored by MET treatment, although TLR4 overexpression abolished the protective effects of MET (Figure 4(h)). Hence, our data concluded that the overactive TLR4/NF-*κ*B signaling disturbed the mitochondria and reprogrammed the glycometabolism to glycolysis represented by PFKFB3. Nevertheless, MET partly corrected the metabolic reprogramming through its inhibitory effects on TLR4/NF-*κ*B.

### 3.4. Pharmacological Concentration of MET Restored the Trophoblastic Redox Homeostasis Partly through the Inhibition of the TLR4/NF-*κ*B Signaling

**Figure 5:**
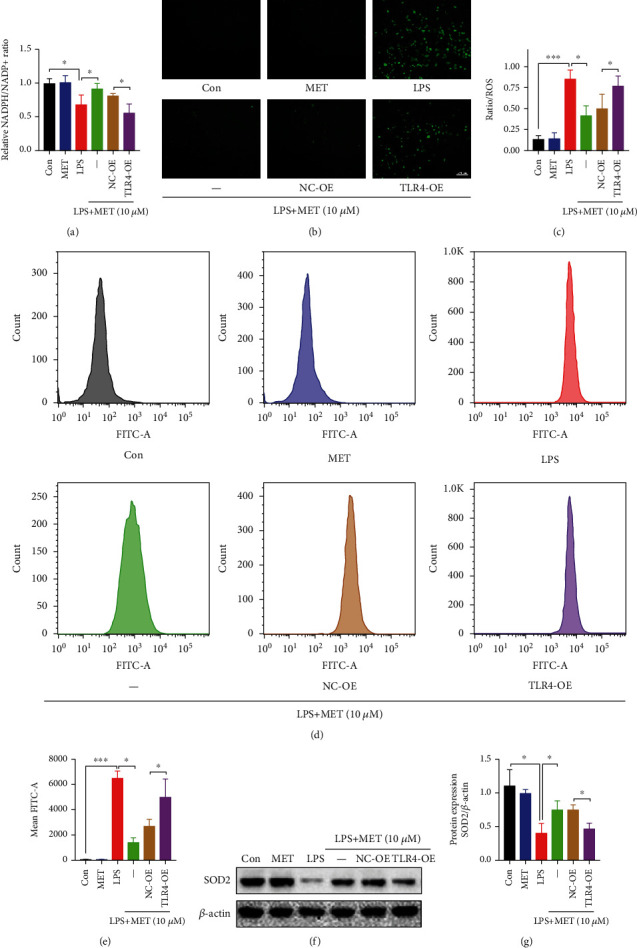
Pharmacological concentration of MET restored the trophoblastic redox homeostasis partly through the inhibition of the TLR4/NF-*κ*B signaling. HTR-8/SVneo cells were transfected with TLR4 plasmid for overexpression or negative vector; 200 ng/ml LPS and 10 *μ*M MET were subsequently incubated. (a) The cellular levels of NADPH were measured in HTR-8/SVneo cells in different groups. (b, c) Representative images showing DCFH-DA fluorescence signals (green) indicative of ROS and the comparison of mean fluorescence intensities across different groups. Scale bar: 100 *μ*m. (d, e) Flow cytometry analysis and quantitative analysis of cellular ROS content in different groups. (f, g) Western blot analysis of SOD2 protein expression and the densitometry quantification. Data are shown as the mean ± SD from three independent experiments. ^∗^*P* < 0.05, ^∗∗^*P* < 0.01, and ^∗∗∗^*P* < 0.001 by Student's *t*-test. SD: standard deviation; NC-OE: negative vector; TLR4-OE: TLR4 overexpression plasmid.

NADPH, the reducing equivalents produced from pentose phosphate pathway, participates in the redox reaction. Therefore, we measured the NADPH/NADP+ ratio, which was diminished by LPS and induced again after MET exposure, though this effect was diminished by TLR4 overexpression again (Figure 5(a)). The immunofluorescence and flow cytometry data showed that LPS significantly increased the ROS levels while MET suppressed ROS generation in LPS-induced trophoblasts, though those were partly reversed via TLR4 overexpression (Figures 5(b)–5(e)). Furthermore, we tested the expression level of SOD2, a vital antioxidant. LPS significantly reduced the SOD2 expression while MET partly elevated the SOD2 expression, though it was decreased by TLR4 overexpression again (Figures 5(f) and 5(g)). Therefore, all these data indicated that pharmacological MET concentration restored the redox homeostasis in LPS-induced trophoblasts partly via the suppression of the TLR4/NF-*κ*B signaling.

### 3.5. Pharmacological Concentration of MET Reduced the Transcription of PFKFB3 via Blocking Transcription Factor NF-*κ*B1 Directly Binding on PFKFB3 Promoter

**Figure 6:**
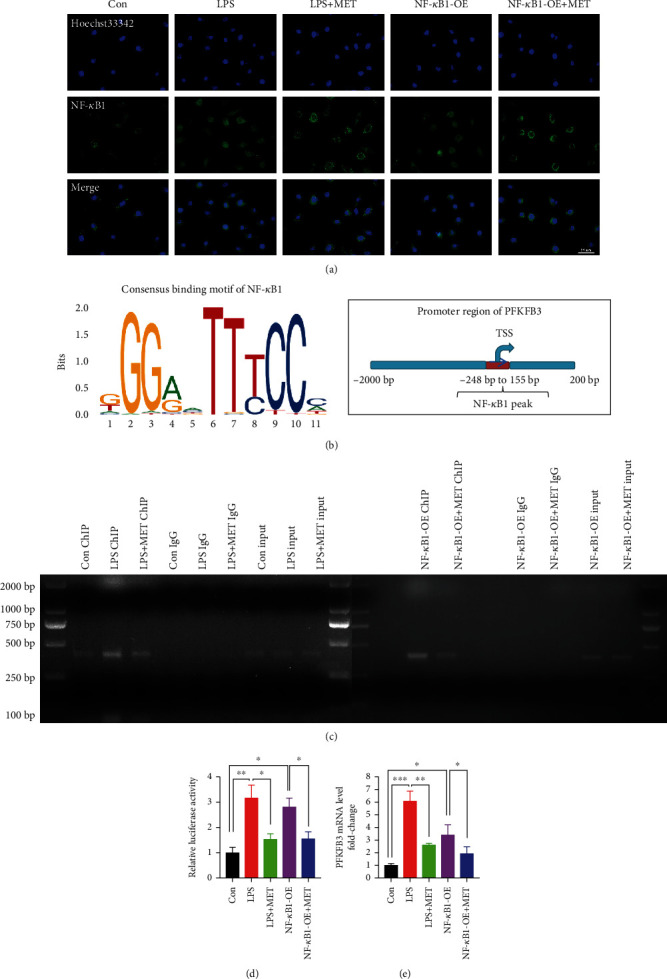
Pharmacological concentration of MET reduced the transcription of PFKFB3 via blocking transcription factor NF-*κ*B1 directly binding on PFKFB3 promoter. HTR-8/SVneo cells were transfected with NF-*κ*B1 plasmid for overexpression; 200 ng/ml LPS and 10 *μ*M MET were subsequently incubated. (a) Representative images of NF-*κ*B1 immunostaining in HTR-8/SVneo cells in different groups. At least 6 images per condition were analyzed. Scale bar: 50 *μ*m. (b) Consensus binding motif of NF-*κ*B1 from the JASPAR database and the NF-*κ*B1 peak in the PFKFB3 promoter were found using the USCS database. (c) In the ChIP assay, PCR products of PFKFB3 in different groups are shown. DNA ChIP-ed with nonspecific IgG was used as a negative control. (d) Dual-luciferase reporter assays were performed to determine the PFKFB3 promoter activity in HTR8/SVneo cells in different groups. (e) qRT-PCR analysis of PFKFB3 mRNA levels in HTR-8/SVneo cells in different groups. Data are shown as the mean ± SD from three independent experiments. ^∗^*P* < 0.05, ^∗∗^*P* < 0.01, and ^∗∗∗^*P* < 0.001 by Student's *t*-test. SD: standard deviation; NF-*κ*B1-OE: NF-*κ*B1 overexpression plasmid.

To explore the underlying mechanism by which inflammatory stimuli induce metabolic reprogramming, we wondered whether the glycolytic enzyme PFKFB3 could be modulated via the NF-*κ*B signaling, a classic inflammatory pathway, at the transcriptional level. Transcription factor NF-*κ*B1 was screened out from factors predicted by the PROMO database in the promoter sequence of PFKFB3 within a dissimilarity margin less or equal than 5% [47, 48]. We validated the efficiency of the NF-*κ*B1 overexpression plasmid after 48 h transfection by western blot analysis (Figure S1B). LPS and NF-*κ*B1 overexpression both enhanced the nuclear translocation of NF-*κ*B1, though they were partly reversed by MET (Figure 6(a)). Then, according to the USCS database [49], we found the peak (from -248 bp to 155 bp relative to TSS) of the transcription factor NF-*κ*B1 in the promotor sequence of PFKFB3. Moreover, according to the JASPAR database [50], we found the binding sites of NF-*κ*B1 in the peak sequence (Figure 6(b)). Therefore, we hypothesized that PFKFB3 might be regulated via the transcription factor NF-*κ*B1. Besides, consistent with the previous reports [30], our abovementioned data also mechanically proved MET an inhibitor of the NF-*κ*B signaling. Thus, MET might decrease NF-*κ*B1 binding on the PFKFB3 promoter. We performed a ChIP assay, showing that the level of NF-*κ*B1 targeting PFKFB3 promoter could be significantly increased on LPS and NF-*κ*B1, which could all be partly reversed by MET (Figure 6(c)). As further confirmation, we cloned the PFKFB3 promoter containing the peak of NF-*κ*B1 to the PGL3.0-Basic reporter plasmid and dual-luciferase reporter assays showed that LPS and NF-*κ*B1 overexpression remarkably upregulated the transcriptional activity of PFKFB3 vectors; however, MET treatment could eliminate these effects (Figure 6(d)). RT-PCR showed that LPS and NF-*κ*B1 overexpression both elevated the mRNA expression of PFKFB3 in trophoblasts, which were partially reversed by MET (Figure 6(e)). All these above data indicated PFKFB3 as a newly identified transcriptional target of the NF-*κ*B signaling, whereas MET reduced the PFKFB3 transcription via blocking transcription factor NF-*κ*B1 binding on the PFKFB3 promoter.

### 3.6. PFKFB3 Regulated the NF-*κ*B Signaling as well as the NLRP3 Inflammasome-Induced Pyroptosis while the Pharmacological Concentration of MET Suppressed Pyroptosis Partly via Inhibiting PFKFB3

**Figure 7:**
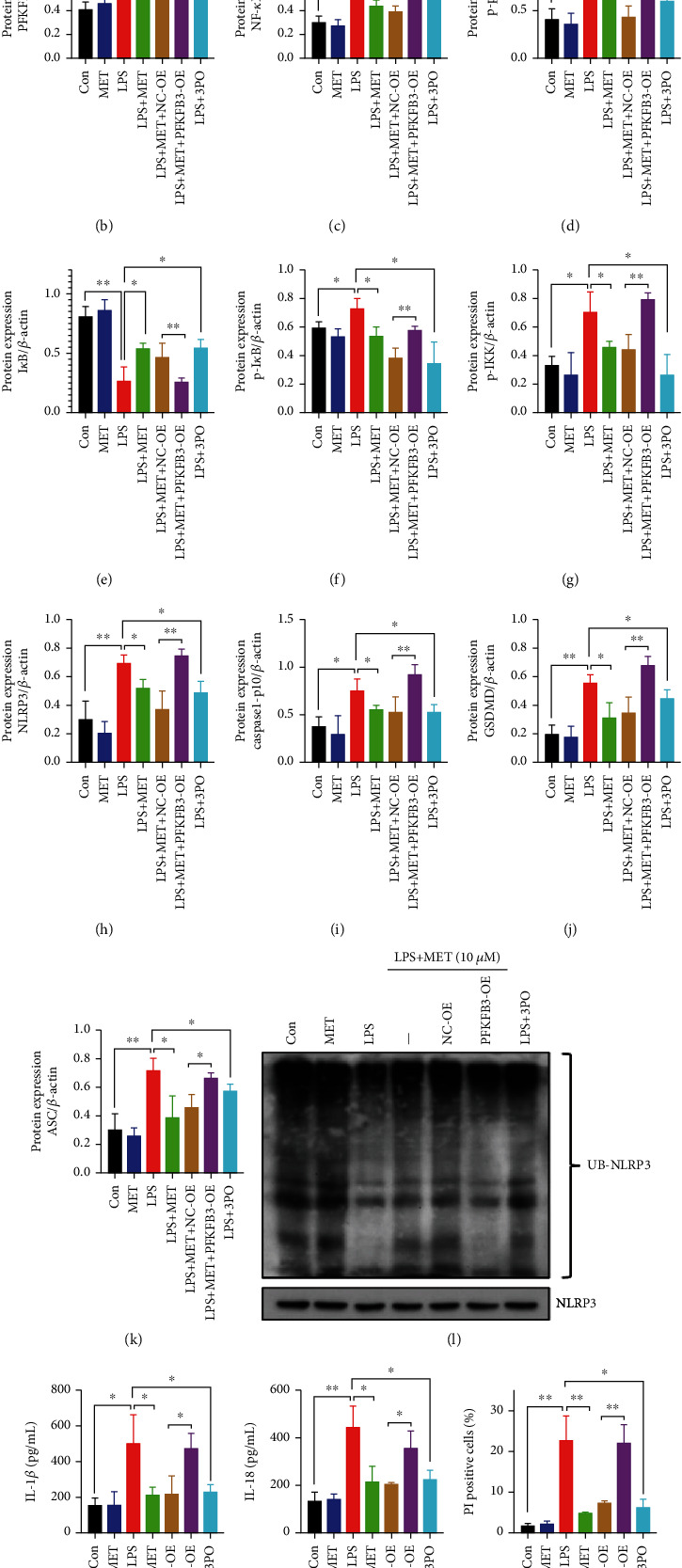
PFKFB3 modulated the NF-*κ*B signaling as well as the NLRP3 inflammasome-induced pyroptosis while the pharmacological concentration of MET suppressed pyroptosis partly via inhibiting PFKFB3. HTR-8/SVneo cells were transfected with PFKFB3 plasmid for overexpression or negative vector; 200 ng/ml LPS, 10 *μ*M MET, and 10 *μ*M 3PO were subsequently incubated. (a–k) Western blot analysis of PFKFB3, NF-*κ*B1, p65, p-p65, I*κ*B, p-I*κ*B, p-IKK, NLRP3, caspase1-p10, GSDMD, and ASC protein expression and densitometry quantification of PFKFB3 (b), NF-*κ*B1 (c), p-p65/p65 (d), I*κ*B (e), p-I*κ*B (f), p-IKK (g), NLRP3 (h), caspase1-p10 (i), GSDMD (j), ASC (k) levels. (l) Analysis of NLRP3 ubiquitination levels in HTR-8/SVneo cells of different groups. (m, n) ELISA analysis of IL-1*β* (m) and IL-18 (n) concentrations in cell culture medium. (o, p) Representative immunofluorescence images and quantification of double-fluorescent staining with PI (red) and Hoechst33342 (blue). Scale bar: 100 *μ*m. Data are shown as the mean ± SD from three independent experiments. ^∗^*P* < 0.05, ^∗∗^*P* < 0.01, and ^∗∗∗^*P* < 0.001 by Student's *t*-test. SD: standard deviation; NC-OE: negative vector; PFKFB3-OE: PFKFB3 overexpression plasmid.

To order to verify whether glycolytic enzyme PFKFB3 participates in the TLR4/NF-*κ*B/NLRP3 inflammasome-induced pyroptosis, we utilized a PFKFB3 inhibitor 3PO [51] at a concentration of 10 *μ*M through CCK8 assays (Figure S1C). Compared with the LPS groups, 3PO inhibited the NF-*κ*B signaling, further decreasing NLRP3, caspase1-p10, ASC, and GSDMD protein levels (Figures 7(a)–7(k)), upregulating the NLRP3 ubiquitination levels (Figure 7(l)), mitigating the IL-1*β* and IL-18 secretion (Figures 7(m) and 7(n)), and reducing the proportion of the PI-stained cells (Figures 7(o) and 7(p)).

We also validated the efficiency of the PFKFB3 overexpression plasmid after 48 h transfection by western blot analysis (Figure S1D). Compared with the NC-OE groups, PFKFB3 overexpression activated the NF-*κ*B signaling again and downregulated the levels of NLRP3 ubiquitination, further increasing the protein expression of NLRP3 as well as those pyroptosis-relevant molecules (Figures 7(a)–7(n)). The analysis of the PI-stained cell also showed a similar trend (Figures 7(o) and 7(p)). Thus, we concluded that glycolytic enzyme PFKFB3 promoted trophoblastic inflammation and NLRP3 inflammasome-induced pyroptosis while MET suppressed those effects partly via inhibiting PFKFB3.

## 4. Discussion

**Figure 8:**
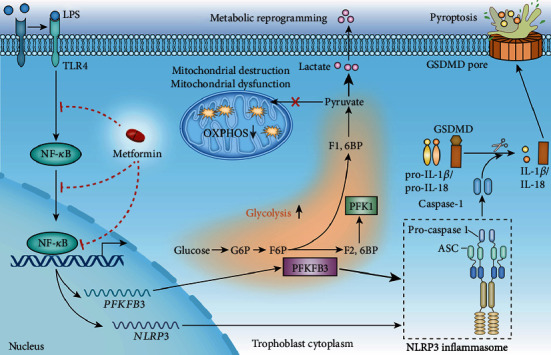
A mechanism diagram. LPS-induced TLR4/NF-*κ*B activation caused NLRP3 inflammasome-induced pyroptosis in trophoblasts. Besides, the TLR4/NF-*κ*B signaling caused mitochondrial destruction and dysfunction; meanwhile, it reprogrammed the glycometabolism to glycolysis with increased PFKFB3 expression. The TLR4/NF-*κ*B signaling induced PFKFB3 expression by transcriptional factor NF-*κ*B1 binding PFKFB3 promoter region. Glycolytic enzyme PFKFB3 further exacerbated NLRP3 inflammasome-induced pyroptosis, leading to positive feedback. Metformin inhibited the TLR4/NF-*κ*B/PFKFB3 signaling to break the vicious circle, expected to be a novel candidate for preeclamptic therapies.

In this study, we found that (1) pharmacological concentration of MET alleviated NLRP3 inflammasome-induced pyroptosis partly through inhibiting the TLR4/NF-*κ*B signaling; (2) pharmacological MET concentration restored the metabolic and redox homeostasis in trophoblasts partly via the suppression of the TLR4/NF-*κ*B signaling; (3) MET decreased the transcription of PFKFB3 via blocking transcription factor NF-*κ*B1 binding on PFKFB3 promoter; (4) PFKFB3 augmented the NF-*κ*B signaling as well as the NLRP3 inflammasome-induced pyroptosis while MET suppressed pyroptosis partly via inhibiting PFKFB3. This study clarifies that the TLR4/NF-*κ*B/PFKFB3 pathway may be a novel link between glucose metabolism reprogramming and NLRP3 inflammasome-induced pyroptosis in trophoblasts. Further, MET inhibits the TLR4/NF-*κ*B/PFKFB3 pathway to ameliorate trophoblastic metabolism disorders and pyroptosis, likely to be a plausible therapy to prevent and cure PE (Figure 8).

LPS, a classic TLR4 agonist, has long been used to make preeclamptic models for years as LPS amplifies the TLR4/NF-*κ*B signaling to trigger trophoblastic inflammation [39, 40]. Consistent with the latest research [37, 52], our results showed that LPS significantly activated the TLR4/NF-*κ*B signaling, further contributing to the NLRP3 inflammasome-induced pyroptosis. Because of the release of the proinflammatory factors IL-1*β* and IL-18, the NLRP3 inflammasome-induced pyroptosis is not only a crucial way to induce trophoblastic death in the preeclamptic placentas but also a possible amplifier of maternal systematic injury via triggering sterile inflammatory cascades. MET has long been found to exert inhibitory effects on the TLR4/NF-*κ*B signaling [30–32]. Therefore, we wonder whether MET can ameliorate trophoblastic pyroptosis. Perhaps because almost all previous studies were conducted with suprapharmacological concentrations (doses) of MET that is much higher than maximally achievable therapeutic concentrations (10~40 *μ*M) with the oral administration of 0.5–2.5 g/d [44], the results of those experiments may be too controversial to truly reflect the effects of MET on trophoblasts [53, 54]. Hence, we carefully chose the pharmacological concentration of MET (10 *μ*M) according to the MET pharmacokinetics during pregnancy [33] and the CCK8 assays in this study. Our data showed that the pharmacological concentration of MET alleviated NLRP3 inflammasome-induced pyroptosis, which partly depended on inhibiting the TLR4/NF-*κ*B signaling. Recently, *in vivo* study has reported that MET exerts protective effects on LPS-induced PE rat models partly through diminishing the NF-*κ*B signaling to ameliorate placental injury, oxidative/nitrative stress, and systemic inflammatory responses [55]. Herein, our study further suggested that MET is beneficial to trophoblasts, which partly lies in the suppression of TLR4/NF-*κ*B-primed pyroptosis.

Up till now, the characteristics of metabolism in the preeclamptic placentas remain ambiguous. A majority of studies have previously reported the mitochondrial swelling and broken cristae together with the mitochondrial dysfunction [56] in the preeclamptic placentas [57], thereby causing the reduced ATP synthesis [58]. But until now, very little research has focused on the glycolytic capacities in the preeclamptic placentas. Metabolomic analysis has found the upregulated concentration of the glycolytic product (lactate and pyruvate) [59, 60] in the serum from PE women, which impaired OXPHOS capacity while enhanced glycolysis in trophoblasts, resulting in a weak ATP synthesis [61] owing to the lower efficiency of glycolysis [20]. PE mice induced by soluble fms-like tyrosine kinase 1 (sFlt-1) showed accelerated glycolytic flux in the placenta labyrinth, together with the ATP shortage [62]. The expressions of glycolytic enzymes PKM2 and PFKFB3 are also abnormally increased in the preeclamptic human placentas [25, 63]. The research mentioned above collectively indicates the plausible metabolic switch to glycolysis in preeclamptic trophoblasts; however, whether it is associated with the TLR4/NF-*κ*B signaling has yet to be confirmed. In our studies, LPS-induced TLR4/NF-*κ*B activation destroyed mitochondrial structure and disrupted mitochondrial function in trophoblasts, which agrees with the latest research [64]. Meanwhile, TLR4/NF-*κ*B activation caused the metabolic reprogramming to the augmented glycolysis, characterized by the elevated secretion of lactate and the increased expression of PFKFB3, the pacemaker of glycolysis. The metabolic reprogramming eventually leads to an ATP shortage in trophoblasts. Currently, it has been reported that MET probably has dual effects on mitochondria, characterized by diminishing impaired mitochondria via mitophagy concomitant with mitochondrial biogenesis to restore mitochondrial function [20]. Furthermore, suprapharmacological MET concentrations (1 mM) disturb mitochondrial respiration while pharmacological MET concentrations (50 *μ*M) improve OXPHOS and mitochondrial function [65, 66]. Analogously, in our study, pharmacological MET concentration (10 *μ*M) ameliorated the mitochondrial dysfunction and damages while suppressed the overactivated glycolysis, thus getting the trophoblastic metabolism back to track with the increased cellular ATP production, which may owe to the inhibitory effect of MET on the TLR4/NF-*κ*B signaling. Consistent with the latest research [46, 67, 68], we also found that pharmacological concentration of MET downregulated the PFKFB3 expression, which is probably the mechanism for MET inhibiting the overactive glycolysis in trophoblasts. Therefore, MET could reverse the TLR4/NF-*κ*B-induced metabolic reprogramming.

The reducing equivalent NADPH is tightly involved in the redox metabolism. In preeclamptic trophoblastic models, the decreased NADPH production symbolized the less diversion of glucose through the pentose phosphate pathway [69], also indicating the increased glycolysis flux as well as the oxidative stress. With the high levels of ROS and lower levels of SOD2 detected in TLR4/NF-*κ*B-activated trophoblasts, our data further elucidated that the TLR4/NF-*κ*B-induced metabolic disorders might inextricably link with the redox disturbances in the PE placentas. Amounts of research have demonstrated that MET can suppress oxidative stress [70]. In vivo study has further demonstrated that MET restored the SOD activity in LPS-induced PE rat models [55]. Also, we have previously reported that PFKFB3 could enhance oxidative stress in LPS-induced trophoblasts [25]. In this study, MET also decreased the TLR4/NF-*κ*B-induced ROS level, which may partly rely on the PFKFB3 inhibition as well as the recuperative NADP+/NADPH ratio and SOD2 level. Therefore, MET may reverse the TLR4/NF-*κ*B-induced metabolic reprogramming and preserved redox homeostasis in trophoblasts.

Previously, we reported that the trophoblastic PFKFB3 expression was significantly upregulated in response to the TLR4 activation [25] while the concrete molecular mechanisms remain unclear. Based on the PFKFB3 promoter sequence, we speculated that transcription factor NF-*κ*B1 is crucial for PFKFB3 expression. Moreover, the nuclear translocation of NF-*κ*B1 in our study was increased once LPS activated or NF-*κ*B1 overexpression; however, those were all reversed by MET. Afterward, dual-luciferase reporter assays and ChIP assays further indicated that LPS stimulation and NF-*κ*B1 overexpression both cause the increased PFKFB3 expression by NF-*κ*B1 directly binding PFKFB3 promoter region while MET decreased the transcription of PFKFB3 via blocking transcription factor NF-*κ*B1 binding on PFKFB3 promoter.

There is accumulating evidence suggesting that cellular metabolic reprogramming to glycolysis closely interacted with the phenotype switch to the proinflammatory states [71]. The abovementioned data showed that the TLR4/NF-*κ*B signaling was an upstream regulator of PFKFB3. However, our previous study has reported that LPS increased the protein expression of PFKFB3 and activated the NF-*κ*B signaling while PFKFB3 knockdown via PFKFB3 siRNA in LPS-induced trophoblasts significantly inhibited the NF-*κ*B signaling [25]. The pharmacological inhibition of PFKFB3 by 3PO in this study also suppressed the NF-*κ*B signaling. Thus, there might be a positive feedback loop between the PFKFB3-activated glycolysis and the NF-*κ*B signaling. Recently, NLRP3 inflammasome was reported to modulate glycolysis by increasing PFKFB3 in macrophages [72]. However, PFKFB3 not only positively modulated the NF-*κ*B signaling but also further activated inflammasome-induced pyroptosis in our study, which attracts us to continue in-depth study in the future as there may exist intricate and feedback relationships between metabolism and inflammatory immune responses. Most of the previous research [73–75] has pointed out that MET can inhibit NLRP3 inflammasome-induced pyroptosis, which owes much to the adenosine monophosphate-activated protein kinase (AMPK), a well-known metabolic sensor monitoring the cellular energy states. Consequently, MET is very likely to regulate cellular pyroptosis via its metabolic modulation effects. In our study, MET alleviated TLR4/NF-*κ*B-stimulated pyroptosis in trophoblasts, which also partly depended on the PFKFB3 inhibition. Ubiquitination is a crucial posttranslational modification of NLRP3 as deubiquitination of NLRP3 is essential for its activation [76]. NLRP3 in resting cells is targeted for ubiquitylation and subsequent proteasomal degradation while LPS primes the NLRP3 inflammasome by inhibiting its ubiquitination and degradation [77]. In this study, LPS significantly reduced the ubiquitination level of NLRP3 while MET partly restore it. However, PFKFB3 overexpression decreased the NLRP3 ubiquitination again. Meanwhile, PFKFB3 inhibitor 3PO significantly upregulated the NLRP3 ubiquitination level in LPS-induced trophoblasts. Thus, all those indicated that PFKFB3 may modulate the NLRP3 inflammasome assembly partly via regulating NLRP3 ubiquitination. However, the underlying mechanism between PFKFB3 and NLRP3 ubiquitination is still not well understood and follow-up experiments are necessary. We clarified that MET suppressed the trophoblastic pyroptosis, which may be partly related to the protection of mitochondrial homeostasis as well as the inhibition of the glycolytic enzyme PFKFB3, thus breaking the vicious circle.

Originally introduced as the first-line therapy for type 2 diabetes mellitus, MET has now been found in clinical trials to significantly reduce the incidence of gestational hypertension and PE in pregnant women with multiple PE-related risks. Additionally, the safety of taking MET during human pregnancies has been preliminarily ensured. Relevant lab studies have demonstrated that the underlying mechanism includes proangiogenic benefits, endothelial protection, anti-inflammatory activities, metabolic modulation, and protective effects on trophoblasts to improve placental development. Together with its easy administration, global availability, and low cost, MET is expected to be a promising option for the prevention and treatment of PE. Nonetheless, there are still some limitations in current studies, and the design of the relevant research scheme is supposed to be further improved in the future. Concretely, experimental MET concentration and PE models should be cautiously chosen. Also, further studies on both short-term and long-term risks and benefits for the fetus are necessary. Furthermore, the clinical trial protocol should be further optimized to evaluate the reduction in the prevalence of PE as a primary endpoint. Therefore, we look forward to more high-quality randomized controlled trials and well-designed lab studies in the future to clarify the place of MET in the prevention and treatment of PE [26].

## 5. Conclusion

Our study verified that the TLR4/NF-*κ*B/PFKFB3 signaling might be a novel link between the reprogrammed glycometabolism and trophoblastic pyroptosis, providing novel insights into the pathogenesis of preeclampsia. However, the pharmacological concentration of MET corrects glycometabolism reprogramming, oxidative stress, and NLRP3 inflammasome-induced pyroptosis partly via inhibiting the TLR4/NF-*κ*B/PFKFB3 signaling in trophoblasts, which further supports that MET is expected to be a promising option for the prevention and treatment of PE.

## Data Availability

The data are included in the article to support the findings of this study.

## Supplementary Materials

Supplementary Materials Supplementary Figure S1: overexpression efficacy identification and CCK8 assay.
