# Supplementary material for: Metformin Corrects Glucose Metabolism Reprogramming and NLRP3 Inflammasome-Induced Pyroptosis via Inhibiting the TLR4/NF-κB/PFKFB3 Signaling in Trophoblasts: Implication for a Potential Therapy of Preeclampsia
Source: Oxid Med Cell Longev. 2021 Nov 11;2021:1806344. doi: 10.1155/2021/1806344 (PMC8601820; doi:10.1155/2021/1806344)
Supplement: Supplementary Materials — Supplementary Figure S1: overexpression efficacy identification and CCK8 assay. [file 1806344.f1.docx]

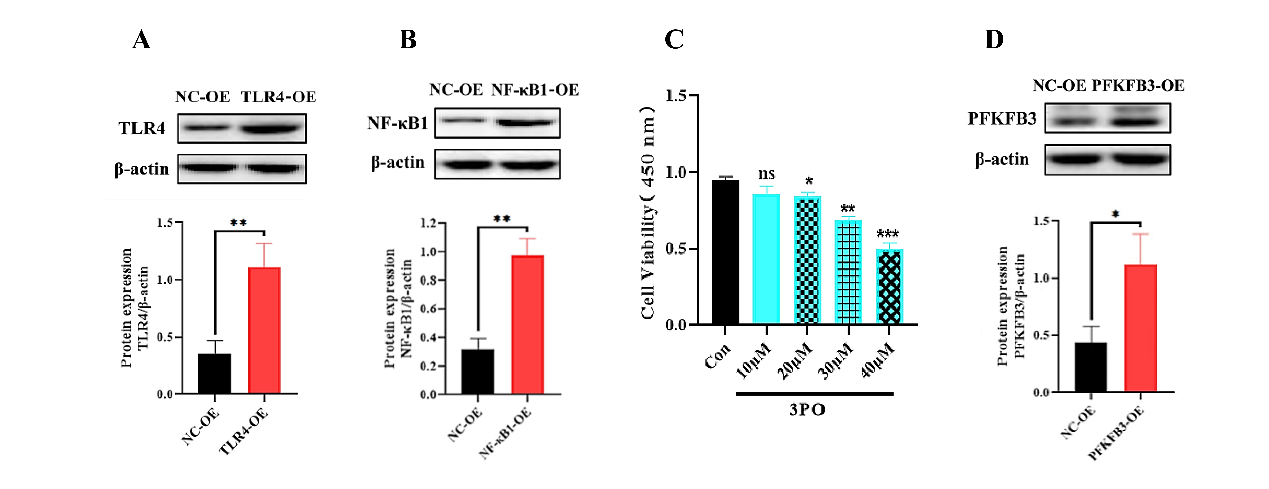


**Figure S1: Overexpression efficacy identification and CCK8 assay. (A)** Western blot analysis and densitometry quantification of TLR4 protein expression in HTR-8/SVneo cells after transfection with vector and the TLR4 plasmid. **(B)** Western blot analysis and densitometry quantification of NF-κB1 protein expression in HTR-8/SVneo cells after transfection with vector and the NF-κB1 plasmid. **(C)** CCK-8 assays of HTR-8/SVneo cells treated with the indicated concentration of 3PO. **(D)** Western blot analysis and densitometry quantification of PFKFB3 protein expression in HTR-8/SVneo cells after transfection with vector and the PFKFB3 plasmid. Data are shown as the mean ± SD from three independent experiments. *: P<0.05. **: P<0.01. ***: P<0.001 by Student’s t-test. SD: standard deviation. NC-OE: negative vector. TLR4-OE: TLR4 overexpression plasmid. NF-κB1-OE: NF-κB1 overexpression plasmid. PFKFB3-OE: PFKFB3 overexpression plasmid.
